# Identification of Epstein-Barr virus BORF2 sequences required for APOBEC3B relocalization

**DOI:** 10.1128/jvi.00693-25

**Published:** 2025-08-21

**Authors:** Farren Clark, Michael A. Carpenter, Reuben S. Harris, Lori Frappier

**Affiliations:** 1 Department of Molecular Genetics, University of Toronto 7938, Toronto, Ontario, Canada; 2 Biochemistry and Structural Biology Department, Howard Hughes Medical Institute, University of Texas Health San Antonio 14742, San Antonio, Texas, USA; University of Virginia, Charlottesville, Virginia, USA

**Keywords:** EBV BORF2, KSHV ORF61, HSV1 UL39, APOBEC3B, SUMO

## Abstract

Epstein-Barr virus (EBV) is a common herpesvirus that establishes lifetime infections in most people worldwide. To protect the lytically replicating EBV genomes from mutation, the EBV BORF2 protein relocalizes the APOBEC3B cytosine deaminase out of the nucleus, sequestering it in cytoplasmic bodies. This property is conserved in BORF2 homologs in other herpesviruses, including Kaposi’s sarcoma-associated herpesvirus ORF61 and herpes simplex virus 1 UL39. Here, we show that a motif conserved in these three proteins (IPAM) is critical for interactions with and relocalization of APOBEC3B. However, the properties of the cytoplasmic bodies formed by BORF2, ORF61, and UL39 differ in that only BORF2 requires APOBEC3B to form cytoplasmic bodies, and only UL39 bodies have properties of aggresomes. We also found that a SUMO-modified site in BORF2 (K741) plays an important role in the formation of bodies with endogenous APOBEC3B, both when expressed on its own and in the context of EBV infection. Additionally, nuclear BORF2-APOBEC3B bodies that formed in early lytic infection contained SUMO, suggesting the importance of SUMOylation in the sequestration of APOBEC3B. Our study provides insights into the mechanisms herpesviruses use to disable APOBEC3B and protect their replicating DNA genomes from undesired editing.

**IMPORTANCE:**

Herpesviruses must protect their replicating DNA genomes from mutation by APOBEC3B, which they do by relocalizing APOBEC3B from the nucleus into cytoplasmic bodies. This is mediated by Epstein-Barr virus BORF2 and its homologs in Kaposi’s sarcoma-associated herpesvirus (ORF61) and herpes simplex virus 1 (UL39). We have shown that a conserved motif in these proteins is critical for this function, and that a SUMO-modified site in BORF2 also plays an important role. This work provides insight into the mechanisms by which BORF2 and its homologs sequester and relocalize APOBEC3B, which is important for maintaining the integrity and infectivity of the respective herpesviruses.

## INTRODUCTION

More than 90% of people worldwide are infected with the γ-herpesvirus Epstein-Barr virus (EBV). EBV plays a causative role in a variety of cancers, including Burkitt’s lymphoma, nasopharyngeal carcinoma, and 10% of gastric carcinoma (1–4), and is also strongly associated with multiple sclerosis (5). Lifetime EBV infections involve alternation between latent and lytic infections. Lytic EBV infection, leading to production of infectious virions, involves expression of approximately 80 viral proteins, many of which appear to manipulate the cellular environment to promote infection and avoid host antiviral responses.

Cells have a variety of defenses against viruses such as EBV, including APOBEC3 enzymes. APOBEC3 proteins are a family of ssDNA cytosine deaminases, two of which (APOBEC3B and APOBEC3A) are oncogenes implicated in a variety of cancers (6). They play an important role in innate immunity by causing hypermutation of the genomes of viruses including HIV, which, without viral countermeasures, can result in a loss of infectivity (7). To defend their genomes from the effects of APOBEC3 enzymes, some viruses encode proteins to counteract these effects (7). A highly studied example is the HIV-1 Vif protein, which recruits a cellular ubiquitin ligase to degrade several APOBEC3 proteins, thereby overcoming their restriction of HIV-1 infection (8).

Using affinity purification-mass spectrometry, our lab previously showed that the EBV BORF2 protein, which serves as the large ribonucleotide reductase (RNR) subunit (R1) of EBV, specifically interacts with APOBEC3B (A3B) (9). In further studies, we showed that BORF2 relocalizes A3B from the nucleus into cytoplasmic bodies, sequestering it away from the viral genomes and protecting replicating EBV DNA from mutation by A3B (9). In the absence of BORF2, EBV genomes became hypermutated by A3B, resulting in loss of infectivity (9). The BORF2-A3B interaction was also found to enable BORF2 to induce G1/S cell cycle arrest, a characteristic of herpesvirus lytic infections (10), through upregulation of p53. The ability to relocalize A3B out of the nucleus also extends to BORF2 homologs in Kaposi’s sarcoma herpesvirus (KSHV; ORF61) and herpes simplex virus 1 (HSV-1; UL39 or ICP6), suggesting the importance of this phenomenon for herpesvirus infections (9, 11, 12). Although headway has been made in understanding the mechanism by which BORF2 binds A3B (9, 13), little is known about the mechanism by which BORF2 and its homologs relocalize A3B into cytoplasmic bodies.

The BORF2 homologs in mouse cytomegalovirus (mCMV) and HSV-1, M45 and UL39, respectively, have been found to bind the receptor-interacting protein kinase 1 (RIPK1), which is important to innate immune signaling, and target it for degradation (14–16). M45 also binds a second innate immune signaling protein, the NFkB essential modulator (NEMO), and targets it for degradation (16). In both cases, M45 and UL39 sequester these proteins in insoluble aggregates to facilitate their degradation by autophagy (16). The formation of these protein aggregates was shown to require a motif named the induced protein aggregation motif, or IPAM, that is conserved in R1 proteins from multiple herpesviruses, including EBV BORF2 and KSHV ORF61 (16). This raises the question as to whether these IPAMs might also play a role in the formation of A3B bodies.

In a previous study, we used a mass spectrometry-based approach to identify SUMO-modified proteins in lytic EBV infection and map the SUMO-modified sites. This revealed that BORF2 is SUMO-modified at lysine 741 (K741) (17). SUMO can have a variety of effects on protein interactions, localization, and function. One of these effects is the non-covalent interaction of SUMO-modified proteins with SUMO-interacting motifs (SIMs) on other proteins, forming an interaction hub. These interactions between SUMOylated proteins and SIMs are essential for the formation of certain cellular bodies, including promyelocytic leukemia (PML) nuclear bodies. However, the impact of SUMOylation of BORF2 on its functions and on the formation of BORF2-A3B bodies has not been examined.

Here, we investigate the requirements for the formation of BORF2-A3B bodies, including the potential role of the BORF2 IPAM and SUMO-modification sites. We show that the IPAM sequence of BORF2 is essential for binding and relocalizing A3B, and that this function of the IPAM is conserved in the BORF2 homologs ORF61 and UL39. However, the nature of the A3B bodies formed by these homologs differs in that only UL39-A3B bodies have properties of aggresomes. Additionally, we found that the K741 SUMO-modified site of BORF2 is important for the formation of bodies with endogenous A3B and that nuclear BORF2-A3B bodies formed early in infection contain SUMO, suggesting a role for SUMOylation in A3B relocalization by BORF2.

## RESULTS

### The BORF2 IPAM is required for A3B binding

**Fig 1:**
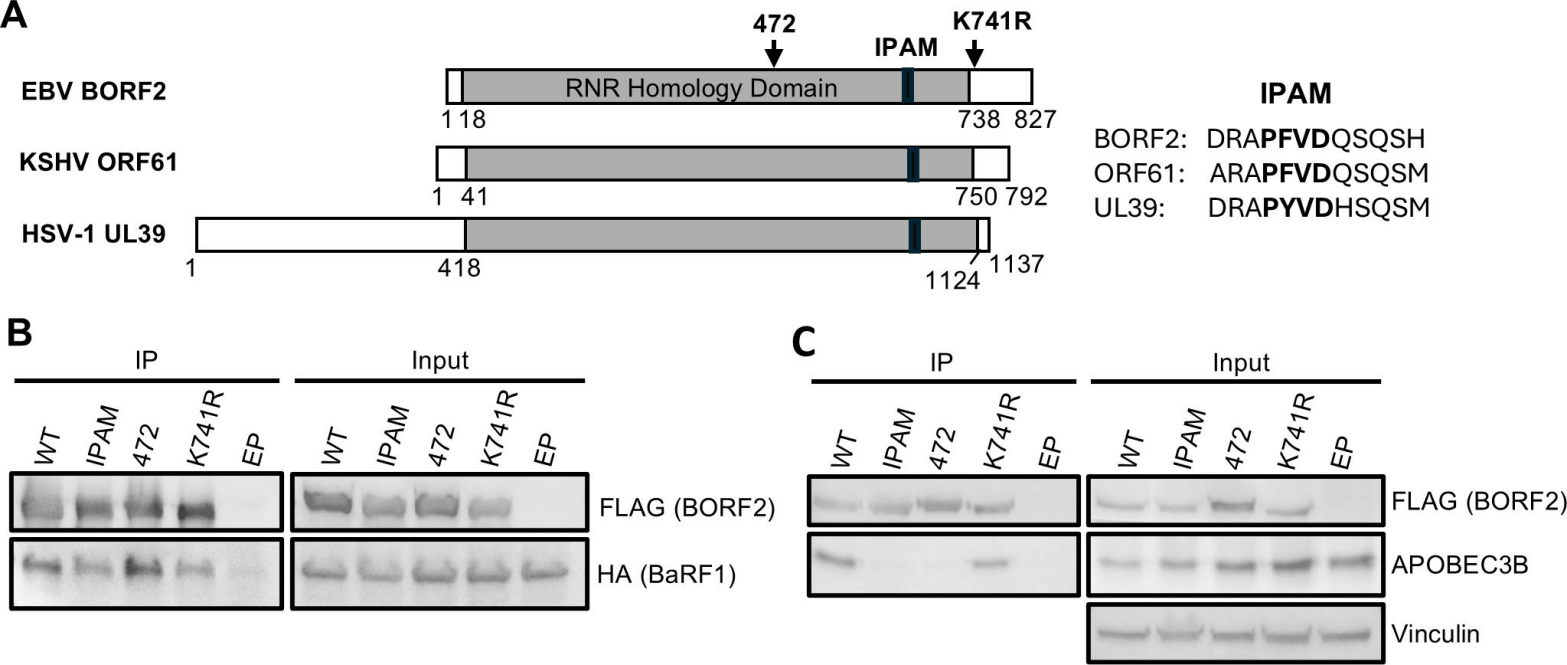
Effect of BORF2 mutations on BaRF1 and APOBEC3B binding. (**A**) Schematic of BORF2, ORF61, and UL39 showing the position of the conserved core ribonucleotide reductase domain (gray) and IPAM sequences (black), in addition to the location of 472 and K741R mutations in BORF2. (**B**) Plasmids expressing wild-type (WT) and mutant FLAG-BORF2 were co-transfected with an HA-BaRF1 expression plasmid in 293T cells, and FLAG immunoprecipitations (IPs) were performed. Western blots of IP and input lysate samples were performed using anti-FLAG and anti-HA antibodies. (**C**) AGS cells were transfected with plasmids expressing WT or mutant FLAG-BORF2, and FLAG IPs were performed. Western blots of IP and input lysate samples were performed using anti-FLAG and anti-A3B antibodies.

We have previously shown that BORF2 binds A3B and relocalizes it from the nucleus to cytoplasmic bodies. While some information on the mechanisms of the BORF2-A3B interaction was determined from a cryo-EM structure of BORF2 bound to the A3B C-terminal domain (13), little is known about the requirements for A3B relocalization and the formation of cytoplasmic bodies. We began to answer these questions by investigating the possible roles of the conserved IPAM sequence (685-PFVD-689), known to mediate some protein interactions in mCMV M45 and HSV-1 UL39 (16), as well as the BORF2 SUMO-modified K741 (17) (Fig. 1A). To this end, we generated expression constructs for FLAG-tagged BORF2 with 685-PFVD-689 mutated to 685-AAAA-689 (IPAM mutant) or with the SUMO-modified K741 mutated to arginine (K741R mutant) to disrupt SUMOylation while retaining the positive charge. We compared these BORF2 mutants to both unmutated BORF2 and the BORF2 472 mutant with a penta-alanine substitution at amino acids 472-476 that was previously shown to be disrupted for A3B binding (9, 10).

BORF2 contains one large folded domain that is conserved among RNR large subunits (Fig. 1A) and binds the small RNR subunit, BaRF1. We first verified that the mutations we generated in BORF2 did not unfold the protein by determining if the BORF2 mutants retained the ability to bind BaRF1. To this end, we expressed FLAG-tagged BORF2 proteins along with HA-tagged BaRF1 and conducted co-immunoprecipitation (IP) experiments. FLAG IPs showed that all the BORF2 mutants recovered HA-BaRF1 at similar levels to wild-type (WT) BORF2 (Fig. 1B), strongly suggesting that they are not misfolded.

We next asked whether the IPAM and K741R mutations in BORF2 affected A3B binding, by expressing FLAG-tagged BORF2 WT, IPAM, K741R, and 472 proteins in AGS gastric carcinoma cells and comparing recovery of endogenous A3B in FLAG IPs. As expected, A3B was recovered with WT but not with 472 BORF2. The K741R BORF2 mutant was found to retain the ability to bind A3B, while the IPAM mutation abrogated A3B binding, indicating a new role for this motif in mediating the A3B interaction (Fig. 1C).

### A3B binding is required for BORF2 body formation

**Fig 2:**
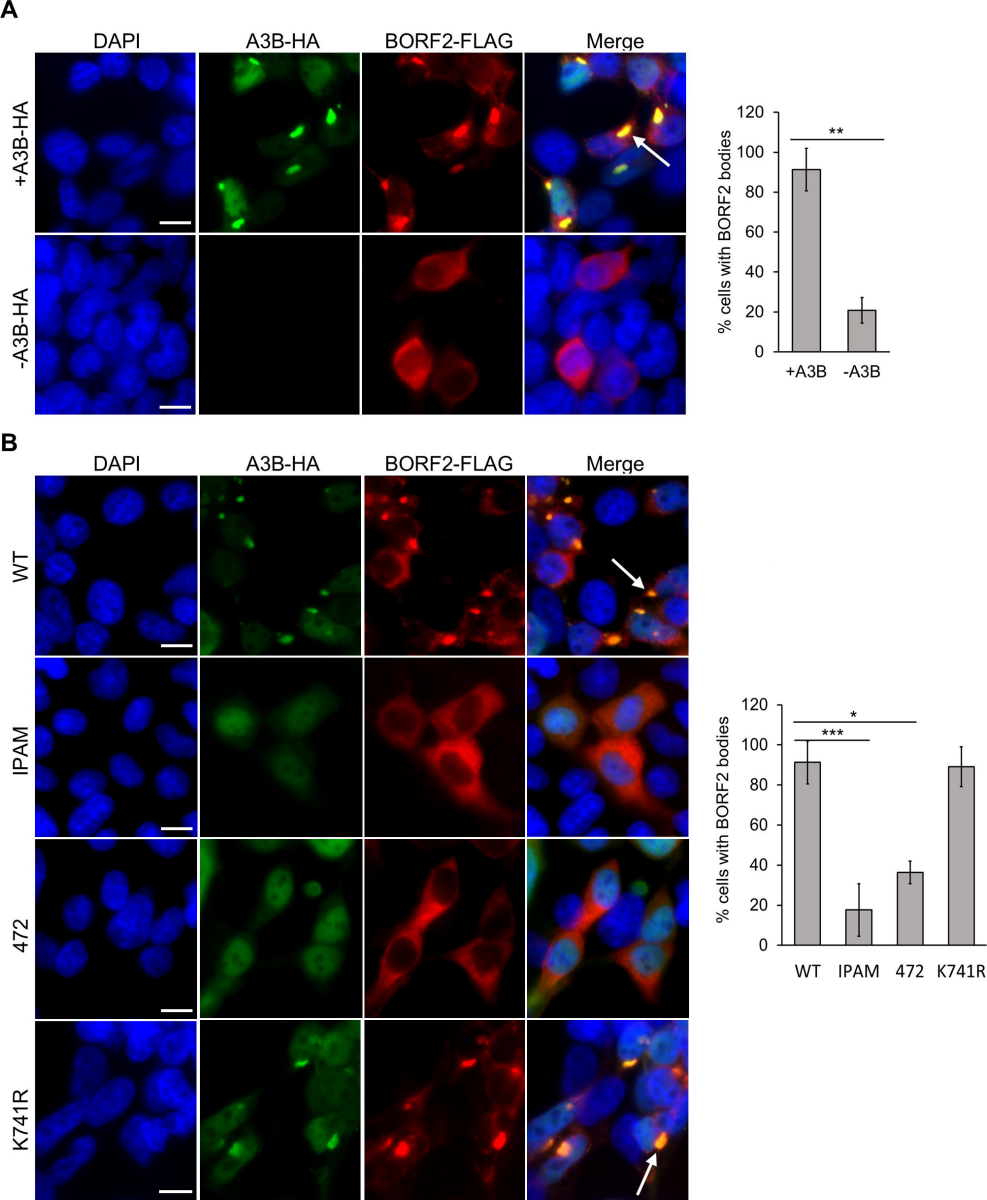
IPAM and 472 BORF2 mutations disrupt body formation with co-expressed A3B. (**A**) 293T cells were transfected with a FLAG-BORF2 expression plasmid, with and without an HA-A3B expression plasmid, then fixed and stained with DAPI and anti-FLAG and anti-HA antibodies. Representative images are shown on the left, where the arrow points to an example of a BORF2-A3B body. One hundred cells expressing BORF2 were scored for the presence or absence of BORF2 bodies. Average values with standard deviation from triplicate experiments are shown on the right. *0.005 < *P* < 0.05, **0.0005 < *P* < 0.005, ***P < 0.0005. (**B**) 293T cells were co-transfected with a plasmid expressing WT or mutant FLAG-BORF2 and a second plasmid expressing HA-A3B. Cells were then fixed, stained, and 100 BORF2-expressing cells scored for whether or not they contained BORF2 bodies as in (A).

We previously showed that BORF2 forms bodies with A3B (9), but the factors involved and the order of events are not fully understood. For example, it is not clear whether BORF2 forms bodies on its own and then recruits A3B, or whether BORF2 can only form bodies when bound to A3B. To test these possibilities, we expressed FLAG-tagged BORF2, with and without HA-tagged A3B, in HEK293T cells. 293T cells were used because they have extremely low levels of endogenous A3B (undetectable with current antibodies); therefore, A3B has to be provided by exogenous expression. Cells were then stained with antibodies against FLAG and HA and imaged by immunofluorescence microscopy. While ~90% of cells expressing both BORF2 and A3B contained prominent co-localized cytoplasmic bodies, cells expressing BORF2 alone showed diffuse cytoplasmic staining with only ~20% containing any more localized bodies (Fig. 2A). Therefore, the presence of APOBEC3B is important for body formation by BORF2.

We then repeated the above experiments with FLAG-tagged BORF2 mutants co-expressed with HA-A3B (Fig. 2B). BORF2 IPAM and 472 mutants, which were disrupted in A3B binding, were impaired in their ability to form bodies with A3B relative to WT BORF2. In contrast, K741R, which retained the ability to bind A3B, formed bodies with A3B at a similar level as WT BORF2. The results support the importance of A3B binding by BORF2 for the formation of BORF2 bodies.

### K741 is required for BORF2 to relocalize and form bodies with endogenous A3B

**Fig 3:**
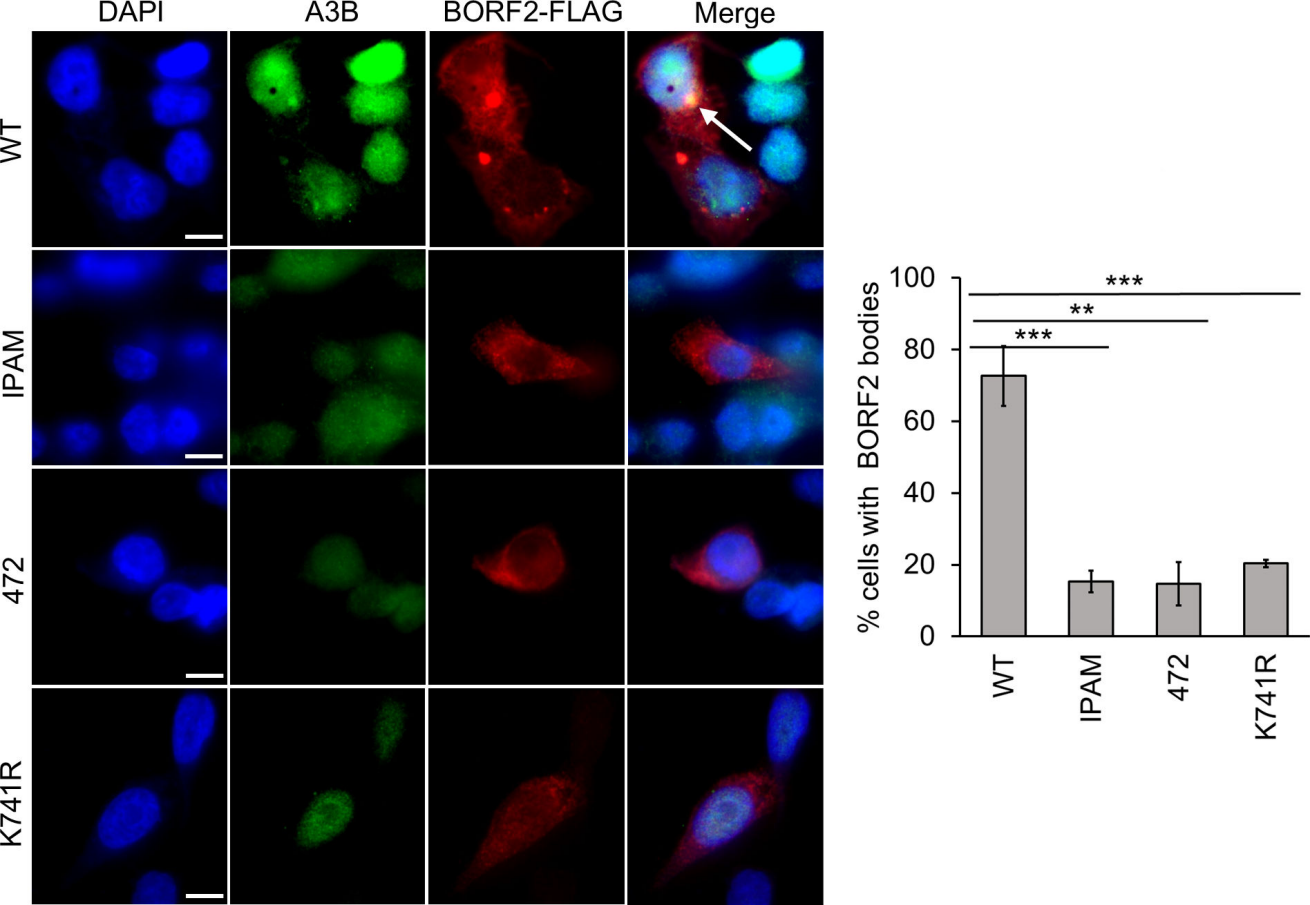
IPAM, 472, and K741R BORF2 mutations disrupt body formation with endogenous A3B. AGS cells were transfected with plasmids expressing WT or mutant FLAG-BORF2, then fixed and stained with DAPI and anti-FLAG and anti-A3B antibodies. Representative images are shown on the left, where the arrow points to an example of a BORF2-A3B cytoplasmic body. Fifty cells expressing BORF2 were scored for whether or not they contained BORF2-A3B bodies. Average values with standard deviation from triplicate experiments are shown on the right. *0.005 < *P* < 0.05, ** 0.0005 < *P* < 0.005, ***P < 0.0005.

While co-expression of BORF2 with A3B demonstrates BORF2’s innate ability to bind A3B and sequester it in cytoplasmic bodies, it does not fully reflect the native situation during infection, in which BORF2 must access A3B in the nucleus and relocalize it to the cytoplasm. To better mimic the physiological condition, we expressed FLAG-tagged BORF2 and BORF2 mutants in AGS cells, which have easily detectable levels of endogenous A3B, then performed immunofluorescence microscopy with antibodies against A3B and FLAG. As expected, BORF2 472 and IPAM mutants, which do not bind or form bodies with co-expressed A3B, were also unable to form bodies with endogenous A3B (Fig. 3). However, BORF2 K741R, which can bind and form bodies with A3B that is co-expressed in the cytoplasm, unexpectedly did not form bodies with or relocalize endogenous, nuclear A3B. This suggests that K741 plays some role in enabling BORF2 to access and interact with endogenous A3B in the nucleus or in relocalizing A3B from the nucleus.

### BORF2-A3B nuclear bodies contain SUMO2 modifications

**Fig 4:**
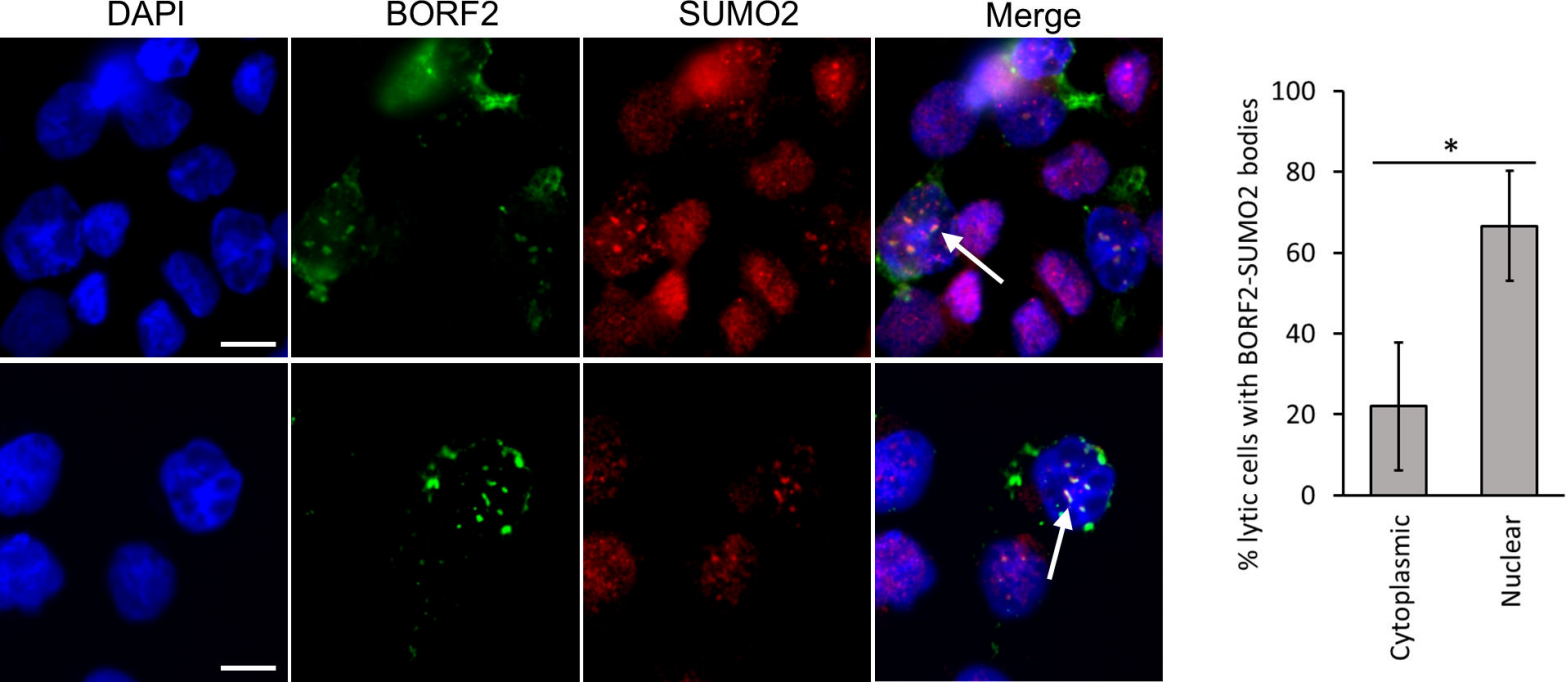
Nuclear BORF2 bodies formed in EBV lytic infection contain SUMOylated proteins. AGS-EBV-Z cells were reactivated to lytic infection with doxycycline treatment, then were fixed and stained with DAPI and anti-BORF2 and anti-SUMO2/3 antibodies. Arrows point to examples of SUMO-staining BORF2 nuclear bodies. The percentage of cells with nuclear or cytoplasmic BORF2 bodies that stained with SUMO antibody was quantified from 50 cells in three biological replicates. Average values with standard deviation for nuclear and cytoplasmic bodies are shown on the right. *0.005 < *P* < 0.05.

BORF2 K741 was identified as being SUMO-modified in EBV lytic infection and is the only BORF2 amino acid found to have this modification (17). Therefore, the importance of K741 in the relocalization of endogenous A3B suggests that this process may involve SUMOylation of BORF2. In the context of EBV lytic infection, we previously showed that, early in infection, BORF2 enters the nucleus and initially relocalizes A3B into nuclear foci (9). This is followed by the formation of BORF2-A3B co-localized bodies outside of the nucleus. To determine whether either of these bodies contains SUMO-modified proteins, we reactivated AGS-EBV-Z cells to the lytic cycle and performed immunofluorescence microscopy using antibodies against BORF2 and SUMO2. We observed that nuclear BORF2 foci have a high frequency of staining with SUMO2 antibody, with ~70% of cells with nuclear BORF2 exhibiting this staining (Fig. 4). Some SUMO2 staining of cytoplasmic BORF2 bodies was also observed, but this was reduced to ~20% of cells with cytoplasmic BORF2 bodies. We also observed some cells that contained both nuclear and cytoplasmic BORF2 bodies, and in these cases, SUMO2 staining was only observed for the nuclear bodies (Fig. 4, bottom panel). These results show that nuclear BORF2-A3B bodies contain SUMO2 modifications that are reduced after relocalization.

### The role of the IPAM in A3B binding and body formation is conserved in KSHV ORF61

**Fig 5:**
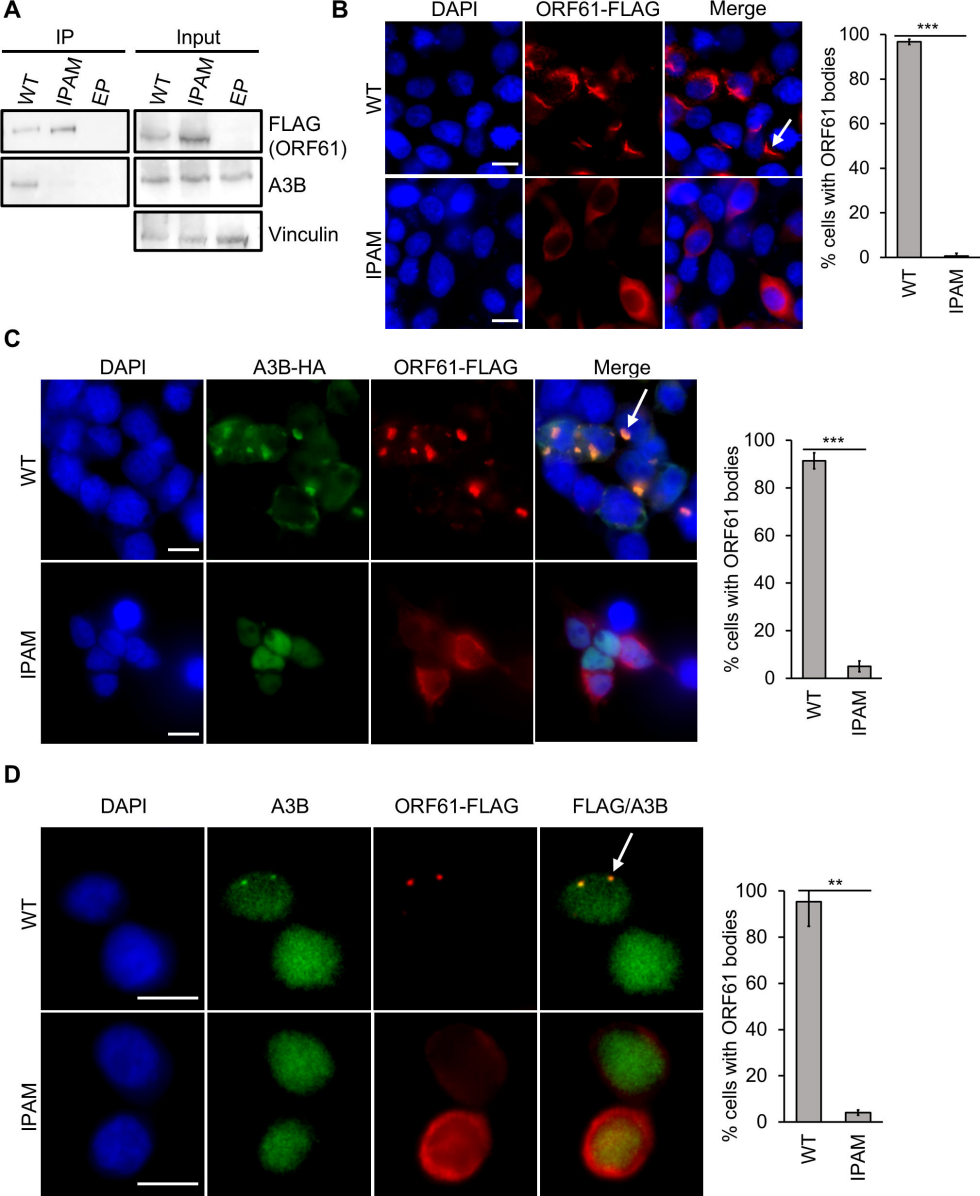
Importance of IPAM in KSHV ORF61 for A3B binding and body formation. (**A**) AGS cells were transfected with plasmids expressing FLAG-ORF61 WT or IPAM mutant, then FLAG IPs were performed. Western blots of IP and input lysate samples were performed using anti-FLAG and anti-A3B antibodies as well as anti-vinculin antibody as a loading control. (**B**) 293T cells were transfected with a plasmid expressing FLAG-ORF61, then fixed and stained with DAPI and anti-FLAG antibody. Representative images are shown on the left, where the arrow points to an example of an ORF61 body. One hundred cells expressing ORF61 were scored for whether or not they contained ORF61 bodies. Average values with standard deviation from triplicate experiments are shown on the right. *0.005 < *P* < 0.05, **0.0005 < *P* < 0.005, ***P < 0.0005. (**C**) 293T cells were co-transfected with a plasmid expressing FLAG-ORF61 WT or IPAM mutant and a second plasmid expressing HA-A3B. Cells were then fixed, stained with DAPI and anti-FLAG and anti-HA antibodies, and 100 ORF61-expressing cells scored for whether or not they contained ORF61 bodies as in (B). The arrow points to an example of an ORF61-A3B body. (**D**) AGS cells were transfected with plasmids expressing FLAG-ORF61 WT or IPAM mutant, then fixed and stained with DAPI and anti-FLAG and anti-A3B antibodies. Representative images are shown on the left, where the arrow points to an example of an ORF61-A3B body. Fifty cells expressing ORF61 were scored for whether or not they contained ORF61-A3B bodies. Average values with standard deviation from triplicate experiments are shown on the right.

Like BORF2, the BORF2 homolog in KSHV, ORF61, binds A3B and relocalizes it into perinuclear bodies (9, 18). Since ORF61 contains the conserved IPAM (16) (Fig. 1A), we mutated it (converting PFVDQ to AAAAA) and asked if this affected the ability of ORF61 to bind and relocalize A3B. Immunoprecipitation of FLAG-tagged ORF61 WT or IPAM mutant from AGS cells showed that only the WT ORF61 immunoprecipitated endogenous A3B (Fig. 5A), indicating the importance of the ORF61 IPAM in binding A3B.

We next examined the ability of ORF61 and ORF61 IPAM to form bodies in the presence and absence of A3B. Unlike BORF2, ORF61 was able to form cytoplasmic bodies in 293T cells, which have undetectable levels of A3B, indicating that ORF61 does not require A3B to form these bodies (Fig. 5B). However, these ORF61 bodies were morphologically distinct from those formed by ORF61 when it was co-expressed with A3B (Fig. 5C), being more elongated than the bodies containing A3B. When the IPAM mutant of ORF61 was used in the same assays, it was found to lack the ability to form bodies either with or without A3B (Fig. 5B and C). This suggests that the ORF61 IPAM mediates interactions in addition to those with A3B, possibly enabling homotypic interactions of ORF61. We also examined whether ORF61 IPAM forms bodies with and relocalizes endogenous A3B in AGS cells. While wild-type ORF61 formed small perinuclear bodies with a fraction of A3B, the IPAM mutant was not observed to form any bodies with A3B (Fig.5D), confirming the importance of the IPAM for ORF61 to form bodies with A3B.

### The role of the IPAM in A3B body formation and relocalization is conserved in HSV-1 UL39

**Fig 6:**
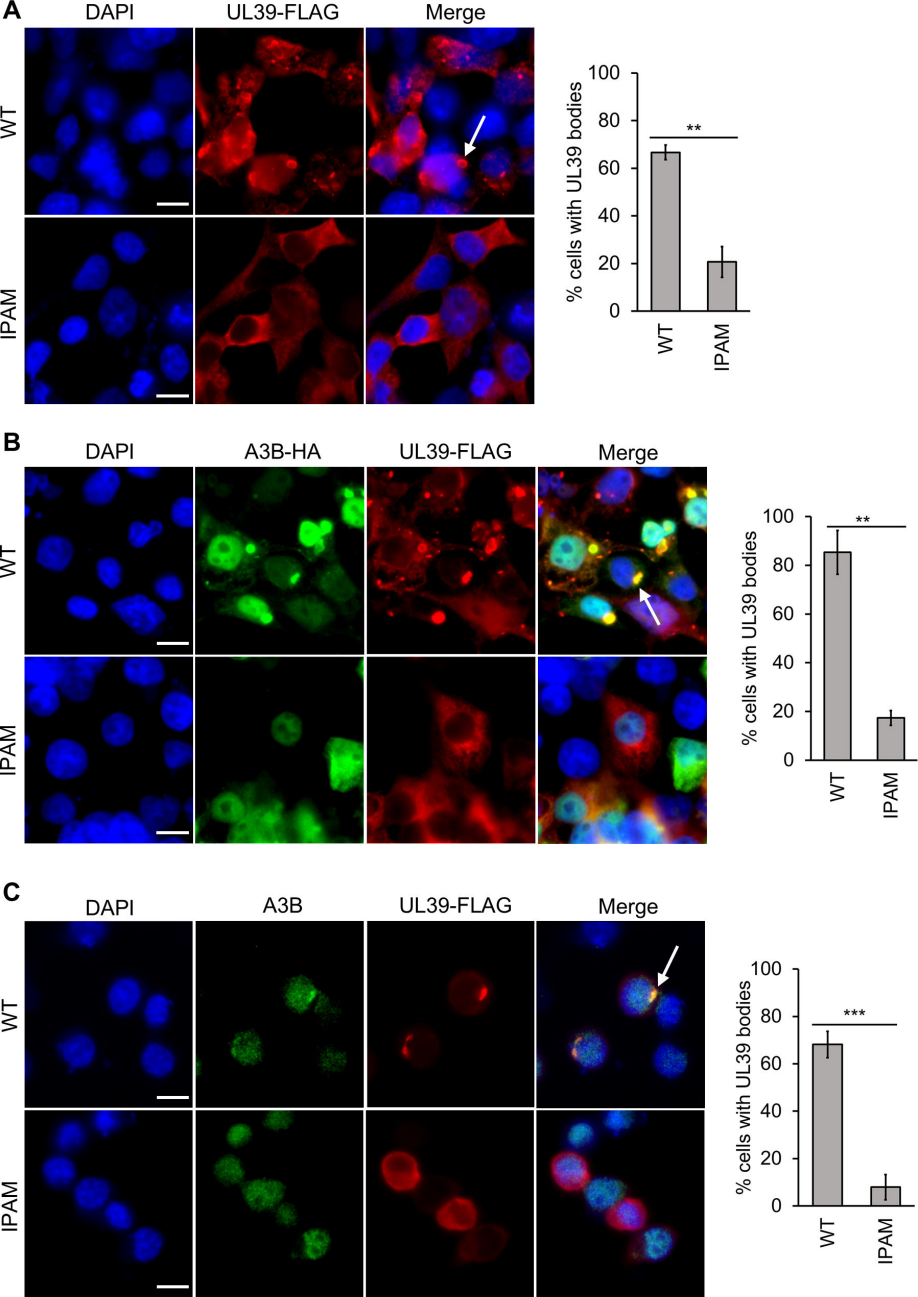
Importance of IPAM in HSV-1 UL39 for UL39-A3B body formation. (**A**) 293T cells were transfected with a plasmid expressing FLAG-UL39, then fixed and stained with DAPI and anti-FLAG antibody. Representative images are shown on the left, where the arrow points to an example of a UL39 body. One hundred cells expressing WT or IPAM mutant FLAG-UL39 were scored for whether or not they contained UL39 bodies. Average values with standard deviation from triplicate experiments are shown on the right. *0.005 < *P* < 0.05, **0.0005 < *P* < 0.005, ***P < 0.0005. (**B**) 293T cells were co-transfected with a plasmid expressing FLAG-UL39 WT or IPAM mutant and a second plasmid expressing HA-A3B. Cells were then fixed, stained, and 100 UL39-expressing cells scored for whether or not they contained UL39 bodies as in (A). The arrow points to an example of a UL39-A3B body. (**C**) AGS cells were transfected with plasmids expressing FLAG-UL39 WT or IPAM mutant, then fixed and stained with DAPI and anti-FLAG and anti-HA antibodies. Representative images are shown on the left, where the arrow points to an example of a UL39-A3B body. Fifty cells expressing UL39 were scored for whether or not they contained UL39-A3B bodies. Average values with standard deviation from triplicate experiments are shown on the right.

The BORF2 homolog HSV-1 UL39 has been found to form bodies with A3B as well as with RIPK1. Body formation with RIPK1 was shown to be dependent on the conserved IPAM (16) (Fig. 1A), but the role of the IPAM in A3B body formation has not been determined. An IPAM mutant of FLAG-tagged UL39 was generated, converting PYVDH to AAAAA, and was compared to WT UL39 for the ability to form bodies in 293T cells in the absence (Fig. 6A) and presence (Fig. 6B) of exogenous HA-A3B. Like ORF61, UL39 formed cytoplasmic structures in the absence of A3B that were dependent on the IPAM (Fig. 6A). Similarly, when co-expressed with A3B, UL39 WT but not IPAM formed cytoplasmic bodies containing A3B (Fig. 6B). Finally, expression of UL39 WT and IPAM mutant in AGS cells revealed that the IPAM was required for the formation of cytoplasmic bodies with endogenous A3B (Fig. 6C). The results indicate the importance of the IPAM in the formation of UL39 bodies with and without A3B, further emphasizing the conserved role of this sequence across multiple RNRs in multiple herpesviruses.

### Unlike UL39 bodies, ORF61 and BORF2 bodies are not aggresomes

**Fig 7:**
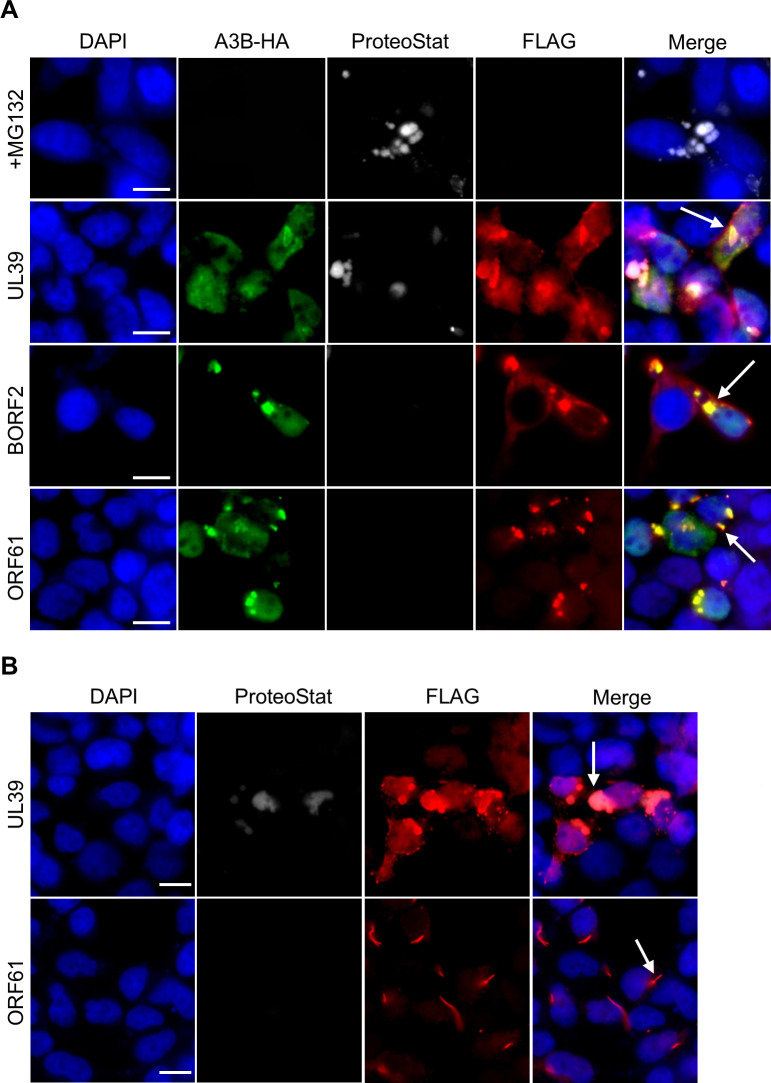
UL39 bodies but not ORF61 or BORF2 bodies are aggresomes. (**A**) 293T cells were co-transfected with a plasmid expressing FLAG-tagged BORF2, ORF61, or UL39 and a second plasmid expressing HA-A3B, then were fixed and stained with DAPI, anti-FLAG and anti-HA antibodies and ProteoStat aggresome detection dye. Arrows point to examples of bodies formed by BORF2, UL39, or ORF61. A positive control for aggresome formation was also performed in which 293T cells were incubated with MG132 for 18 hours (top panel). (**B**) 293T cells were transfected with plasmids expressing FLAG-ORF61 or FLAG-UL39, then cells were fixed and stained as in (A).

IPAM-dependent UL39 and M45 bodies that formed with RIPK1 and NEMO have been previously characterized as aggresomes using an aggresome-detection dye (16). Therefore, we examined whether the IPAM-dependent bodies formed by BORF2, ORF61, or UL39 with A3B were aggresomes. To this end, FLAG-tagged BORF2, ORF61, or UL39 were co-expressed with HA-A3B in 293T cells, followed by staining with anti-FLAG and HA antibodies and ProteoStat aggresome detection dye. Treatment of untransfected 293T cells with the MG132 proteasomal inhibitor served as a positive control for aggresome induction (Fig. 7A, top panel). Immunofluorescence imaging showed that UL39-A3B bodies, but not BORF2-A3B or ORF61-A3B bodies, were stained by ProteoStat, indicating that only the UL39-A3B bodies were aggresomes (Fig. 7A). We also examined ORF61 and UL39 bodies that formed in the absence of A3B and found that UL39 but not ORF61 bodies stained with ProteoStat (Fig. 7B). These results suggest that bodies formed by UL39, with or without A3B, are aggresomes, while those formed with BORF2 and ORF61 are not. Therefore, IPAM plays important roles in the formation of multiple types of cytoplasmic bodies.

### Effects of BORF2 mutations on BORF2-A3B body formation in EBV lytic infection

**Fig 8:**
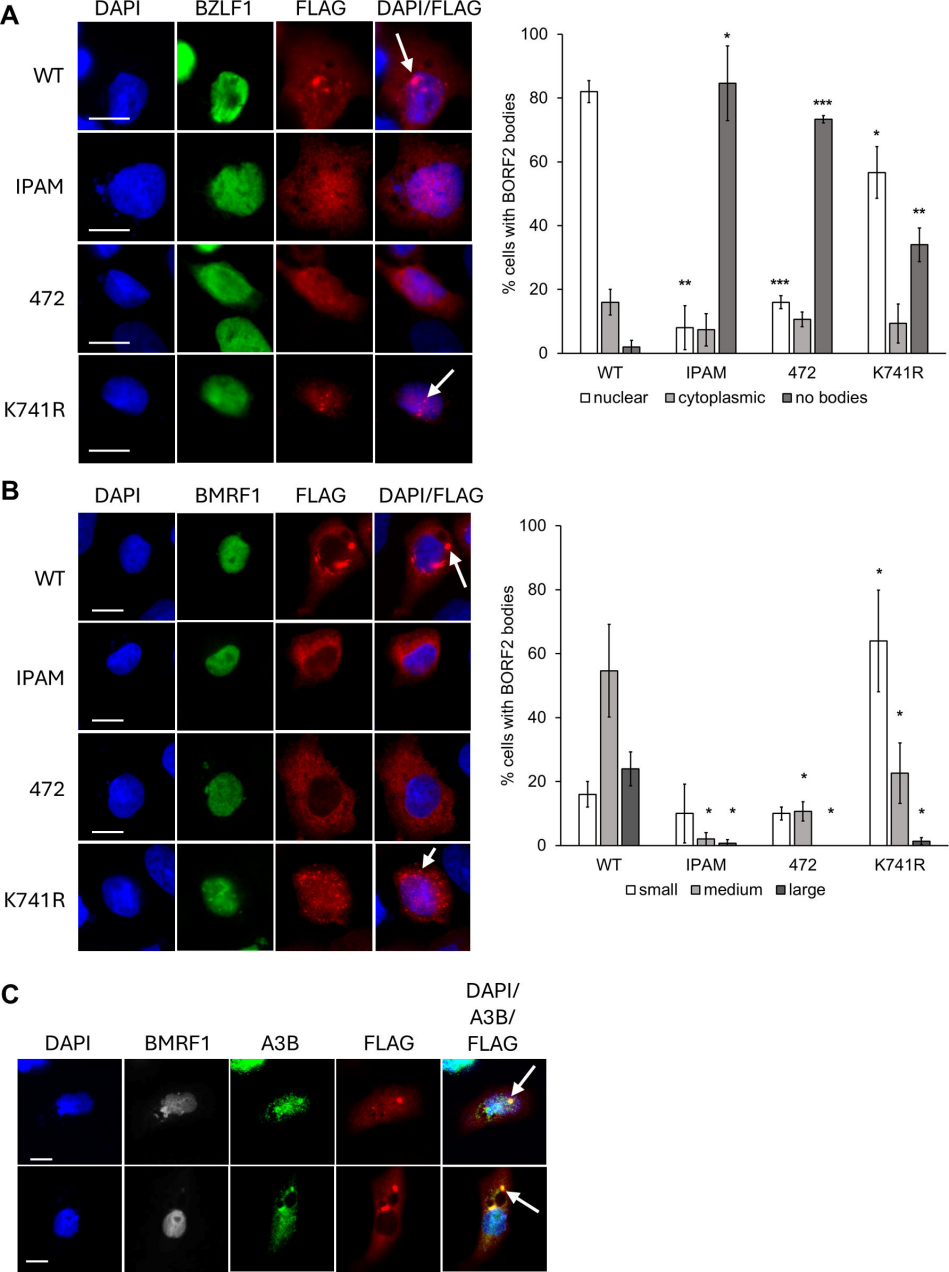
IPAM and K741R BORF2 mutations impair the formation of nuclear and cytoplasmic BORF2-A3B bodies in lytic infection. (**A**) AGS-EBV-BORF2KO cells were transfected with a plasmid expressing FLAG-tagged WT or mutant BORF2 and a plasmid expressing BZLF1 to induce the lytic cycle, then were fixed 18 hours later and stained with DAPI and antibodies against FLAG and BZLF1. Representative images are shown on the left, where arrows point to example of BORF2 nuclear bodies. Fifty cells expressing FLAG-BORF2 and BZLF1 were scored for whether or not they contained nuclear bodies, only cytoplasmic bodies, or no BORF2 bodies. Average values with standard deviation from triplicate experiments are shown on the right, with significance shown relative to WT BORF values. *0.005 < *P* < 0.05, **0.0005 < *P* < 0.005, ***P < 0.0005. (**B**) The same experiment as in (A) except cells were reactivated for 24 hours then stained with DAPI and antibodies against FLAG and BMRF1. Representative images are shown on the left, where the arrows indicate cytoplasmic BORF2 bodies . Fifty cells expressing FLAG-BORF2 and BMRF1 were scored for whether they contained large bodies (with or without medium or small bodies; black bars), medium bodies (with or without small bodies; gray bars), or only small bodies (white bars). Average values with standard deviation from triplicate experiments are shown on the right with significance shown relative to WT BORF values. *0.005 < *P* < 0 .05, **0.0005 < *P* < 0.005. (**C**) AGS-EBV-BORF2KO cells were transfected with a plasmid expressing WT FLAG-BORF2 and a plasmid expressing BZLF1, then were fixed 24 hours later and stained with DAPI and antibodies against FLAG, BMRF1, and A3B. Representative images of cells with BORF2 nuclear (top row) and large cytoplasmic (bottom row) bodies are shown and examples are indicated by the arrows.

We previously showed that, in EBV lytic infection, BORF2 forms nuclear bodies with A3B early in infection and cytoplasmic bodies later in infection (9). We also generated EBV with a knockout of BORF2 in the AGS-EBV cells and showed that this abrogated the formation of both nuclear and cytoplasmic A3B bodies (9). To determine how the BORF2 mutations affect the formation of these bodies in infection, we transfected AGS cells containing the BORF2 knockout EBV with plasmids expressing BORF2 WT or mutants and with a plasmid expressing BZLF1 to activate lytic infection. We then examined cells expressing BZLF1 and FLAG-BORF2 for BORF2 bodies. Control experiments showing specificity of the BZLF1 antibody are shown in Fig. S1A. At 18 hours post-reactivation, we observed that ~80% of the cells expressing WT BORF2 and BZLF1 had BORF2 nuclear bodies, whereas BORF2 bodies (nuclear or cytoplasmic) were rarely seen with the 472 or IPAM BORF2 mutants (Fig. 8A). For BORF2 K741R, there was a reduction in the number of cells with nuclear bodies; but most strikingly, these bodies were much smaller and less intense than for WT BORF2 (Fig. 8A, bottom row).

At 24 hours post-reactivation, virtually all of the cells containing WT BORF2 that had progressed into the lytic cycle (as determined by staining for the early protein, BMRF1; see control experiments for specificity of the BMRF1 antibody for lytic cells in Fig. S1B) contained cytoplasmic BORF2 bodies of various sizes. In contrast, cytoplasmic bodies of any kind were rarely seen in lytic cells expressing the IPAM or 472 BORF2 mutants, while lytic cells expressing the K741R BORF2 predominantly had only small BORF2 bodies that were less intense than for WT BORF2 (Fig. 8B, bottom row). In addition, while large WT BORF2 bodies (nuclear and cytoplasmic) stained for A3B as we have shown previously (9) (Fig. 8C), due to the low sensitivity of the A3B antibody, we have not been able to confirm if the small-sized and medium-sized BORF2 bodies that form in EBV infection contain A3B. Our results confirm the importance of both IPAM and K741 for the formation of nuclear and cytoplasmic BORF2-A3B bodies in EBV lytic infection.

## DISCUSSION

BORF2 and its homologs in KSHV (ORF61) and HSV-1 (UL39) relocalize A3B out of the nucleus and into cytoplasmic bodies, protecting the replicating viral genomes in the nucleus from A3B mutation. In this study, we discovered that the specific BORF2 sequence K741, which is its only known SUMO-modified residue, plays a crucial role in this process. Additionally, we found that the IPAM sequence, conserved in BORF2, ORF61, and UL39, is critical for A3B interactions and relocalization.

The IPAM was first described in mCMV M45 and HSV-1 UL39 by Muscolino et al. (16) as important for interactions with RIPK1 and NEMO. Since the IPAM is also conserved in BORF2 and ORF61, we investigated its importance for interactions with A3B. We found that this motif plays an important role in BORF2 and ORF61 binding to A3B and, consistent with this finding, IPAM mutants of BORF2 and ORF61 were disrupted in the ability to relocalize A3B into cytoplasmic bodies. Similarly, UL39 lost the ability to form cytoplasmic bodies with A3B when the IPAM sequence was mutated. While our manuscript was in review, Luoto et al. (19) reported that mutation of the IPAM in KSHV ORF61 disrupted its ability to interact with and relocalize A3B, in excellent agreement with our results. The study by Muscolino et al. (16) suggested that the contribution of the M45 IPAM to the formation of M45-RIPK1 aggregates involved the propensity of M45 to dimerize and form higher-order oligomers. In keeping with that model, we observed that ORF61 and UL39 could form cellular bodies in the absence of A3B that were dependent on the IPAM sequence (Fig. 5B and 6A). This suggests that, in ORF61 and UL39, the IPAM is important for higher-order homotypic interactions or for heterotypic interactions in addition to those with A3B.

In contrast to ORF61 and UL39, BORF2 does not form bodies in the absence of A3B (Fig. 2A), and we found no evidence that the IPAM inhibits its dimerization, as determined by the ability of IPAM mutants with different epitope tags to immunoprecipitate each other (data not shown). This aligns with a previous cryo-EM structure of BORF2 bound to A3B-CTD, which shows that, unlike other large ribonucleotide reductase subunits, BORF2 dimerizes through its N-terminal region and does not involve the IPAM (13). However, the IPAM in BORF2 is critical for binding A3B, as evidenced by the inability of the IPAM mutant to immunoprecipitate A3B or sequester it into bodies. The cryo-EM structure of BORF2 bound to A3B-CTD did not identify interactions mediated by the IPAM (13), but there could be several reasons for this discrepancy. First, cryo-EM captures the protein in only one conformation, and there may be additional conformations of the BORF2 interaction with A3B-CTD, in which the IPAM makes contacts with A3B. Secondly, the IPAM mutations may affect A3B binding indirectly by altering the conformation of A3B binding regions in BORF2. Thirdly, since the cryo-EM was done using A3B-CTD as opposed to the whole protein, it may be that the presence of the NTD alters the BORF2 interaction, by changing the conformation of the CTD or by providing additional contact with the NTD.

The bodies that mCMV M45 and UL39 form with RIPK1 through interactions with their IPAM sequence are thought to be aggresomes, based on their co-staining with the ProteoStat aggresome detection dye, commonly used to detect protein aggregates (16). The importance of the IPAM in the formation of BORF2/ORF61/UL39-A3B bodies prompted us to ask if these bodies were also aggresomes. Consistent with the findings of Muscolino et al. (16), we found that bodies formed by UL39 on its own or in complex with A3B stained with ProteoStat and therefore contained protein aggregates. However, the BORF2-A3B bodies and the bodies ORF61 formed with A3B and without A3B were not stained by ProteoStat, indicating that they differ from bodies containing UL39 and are unlikely to be aggresomes. These findings are consistent with the fact that, unlike UL39-RIPK1 bodies that facilitate the degradation of RIPK1 by autophagy (16), BORF2 promotes the stabilization rather than the degradation of A3B (9). In addition, while the ProteoStat staining indicates that the UL39-A3B bodies contain protein aggregates, we have not seen evidence that UL39 induces A3B degradation. We conclude that the IPAM can mediate a variety of protein interactions that result in the formation of different types of cellular bodies.

In a previous SUMO proteomics study conducted in EBV infection, we identified one SUMO-modified site in BORF2 mapping to K741 (16). Our current study shows that BORF2 with a K741R mutation retains the ability to bind A3B, indicating that SUMOylation of BORF2 is not required to bind A3B. Consistent with this finding, we found that when BORF2 K741R is co-expressed with A3B, it can sequester it in cytoplasmic bodies (Fig. 2B). However, the BORF2 K741R mutant was not able to form bodies with or relocalize endogenous nuclear A3B (Fig. 3). When the K741R mutant was expressed in the context of EBV lytic infection, it also exhibited prominent defects in forming bodies as compared to WT BORF2. At early infection times when BORF2 forms nuclear bodies with A3B (10), nuclear bodies observed for BORF2 K741R were consistently smaller than for BORF2 (Fig. 8A). At later times when BORF2 forms prominent perinuclear bodies with A3B, again we predominantly observed only much smaller bodies formed by K741R (Fig. 8B). Together, the results indicate that, although the K741R mutant retains the ability to bind A3B, K741 of BORF2 plays an important role in the formation of bodies with nuclear A3B, thus affecting its relocalization.

Since K741 is SUMO-modified, the results suggest a role for SUMOylation in the formation of BORF2-A3B bodies. In support of this hypothesis, the majority of early nuclear BORF2 bodies stained with a SUMO2/3 antibody (Fig. 4), indicating the presence of SUMO-modified proteins within the nuclear bodies, which could include BORF2. SUMO modifications are well known for playing roles in the formation of some cellular bodies, including PML nuclear bodies, whose formation depends on SUMO modifications of PML proteins and their binding SUMO-interacting motifs in PML and other proteins (20–22). We speculate that, similar to PML bodies, the initial formation of nuclear BORF2-A3B bodies may involve a scaffold of SUMO-SIM interactions involving BORF2 and additional proteins.

In summary, we have identified residues in BORF2 that are critical for the sequestration of A3B in cytoplasmic bodies that protect lytically replicating EBV genomes from A3B mutation, and identified similarities and differences in A3B sequestration by BORF2 homologs in KSHV and HSV-1. Further studies are needed to more completely understand the composition of the BORF2-A3B bodies and their nuclear to cytoplasmic transition.

## MATERIALS AND METHODS

### Cell lines

HEK293T cells were cultured in Dulbecco’s modified Eagle medium. AGS gastric carcinoma cells, EBV-positive AGS cells (AGS-EBV), AGS with BORF2-null EBV (AGS-EBV-B2KO) (9), and AGS-EBV with a Tet-inducible BZLF1 (AGS-EBV-Z) (23) were cultured in RPMI. All media were supplemented with 10% heat-inactivated fetal bovine serum and 1× penicillin-streptomycin. Media for AGS-EBV-Z and AGS-EBV-B2KO also contained 80 µg/mL G418 (geneticin).

### Plasmids

The pcDNA3.1 (+) APOBEC3B-HA (24) (GenBank accession number: NM_004900), the pCMV3F constructs expressing BORF2, HSV-1 UL39, and KSHV ORF61 (10), the pBS-Zta (BZLF1) (25), and the pcDNA4-BORF2472-3xFLAG mutant in which amino acids 472-477 were converted into AAAAA (10) have been previously described. The BORF2 K741R mutant was generated in pCMV3F-BORF2 by site-directed mutagenesis using Phusion High-Fidelity DNA Polymerase (Thermo Scientific) with forward primer 5′-GGTGATGGAGTGTAGGGCCAGCGCGGCTC-3′ and reverse primer 5′-GAGCCGCGCTGGCCCTACACTCCATCACC-3′. The BORF2 IPAM mutant in which amino acids 685-PFVD-689 were converted to AAAA was generated in pCMV3F-BORF2 by site-directed mutagenesis using PfuTurbo DNA Polymerase (Agilent) with forward primer 5′-GCCGCCGCTGCGGCCCAGAGCCAATCTCACAGCCTG-3′ and reverse primer 5′-CTGGGCCGCAGCGGCGGCCCTGTCCCGGGACATC-3′. The HSV-1 UL39 IPAM mutant in which amino acids 1069-PYVDH-1073 were converted to AAAAA was generated in pCMV3F-UL39 (10) by site-directed mutagenesis using PfuTurbo DNA Polymerase with forward primer 5′-TGTGTGCGGACCGCGCCGCCGCCGCCGCCGCTAGCCAATCCATGACCC-3′ and reverse primer 5′-GGGTCATGGATTGGCTAGCGGCGGCGGCGGCGGCGCGGTCCGCACACA-3′. The KSHV ORF61 IPAM mutant in which amino acids 697-PFVDQ-702 were converted to AAAAA was generated in pCMV3F-ORF61 (10) by site-directed mutagenesis using PfuTurbo DNA Polymerase with forward primer 5′-GCTCGTGCCAGGGCGGCGGCTGCAGCCGCGAGCCAGTCCATGAGC-3′ and reverse primer 5′-GCTCATGGACTGGCTCGCGGCTGCAGCCGCCGCCCTGGCACGAGC-3′.

### Immunoprecipitation

HEK293T cells at 70% confluence were co-transfected with 4 µg of pcDNA-A3Bctd and either 4 µg of pCMV3F-BORF2 IPAM, 4 µg pCMV3F-BORF2 K741R, pcDNA-BORF2 472, pCMV3F, or 2 µg pCMV3F-BORF2 WT using linear polyethylenimine (Polysciences, Inc.) according to the manufacturer’s recommendations. AGS cells at 70% confluence were transfected with 12 µg of pCMV3F-BORF2 IPAM, pCMV3F-BORF2 K741R, pcDNA-BORF2 472, pCMV3F, or pCMV3F-ORF61 IPAM, or 6 µg of pCMV3F-BORF2 using linear polyethylenimine. After 24 hours (HEK293T cells) or 48 hours (AGS cells), cells were harvested, washed in phosphate buffered saline (PBS), resuspended in 300 µL lysis buffer (150 mM NaCl, 50 mM Tris-HCl, 0.5% NP-40), vortexed, and sonicated for 20 pulses. Lysates were clarified by centrifugation and mixed with 10 µL anti-FLAG M2 magnetic beads at 4°C with gentle rotation overnight. The beads were collected by centrifugation and washed five times with lysis buffer. Proteins were recovered by boiling in 20 µL of SDS loading buffer for 10 minutes. The sample was analyzed by 12% SDS-PAGE (for WT and mutant BORF2) or 10% SDS-PAGE (for WT and mutant ORF61) and transferred to a nitrocellulose membrane (BioRad) for Western blotting.

### Western blotting

Nitrocellulose membranes were incubated in 4% bovine serum albumin (BSA) in PBS overnight at 4°C, then with primary antibody in 2% BSA and 0.5% Tween 20 in PBS for 1 hour at room temperature (for mouse anti-FLAG M2; Sigma-Aldrich; F1084; 1:10,000) or overnight at 4°C (for rabbit anti-HA [Cell Signaling Technology; C29F4; 1:2,000], rabbit anti-A3B [26]; 1:1,000, mouse anti-beta actin [Invitrogen; BA3R; 1:5,000], and mouse anti-vinculin [Santa Cruz Biotechnology; sc-73614; 1:5,000]). Blots were then rinsed three times with 1% Tween 20 in PBS and incubated with secondary antibodies goat anti-rabbit horseradish peroxidase (HRP) (Sigma-Aldrich; SAB3700878; 1:5,000) or goat anti-mouse HRP (Sigma-Aldrich; SAB3701066; 1:5,000) for 1 hour at room temperature. Blots were washed five times with 1% Tween 20 in PBS, and the protein bands were visualized using Western Blotting Luminol Reagent (ImmunoCruz), Clarity Western ECL Substrate (Bio-Rad), or ECL Prime Western Blotting Detection Reagent (Amersham), and a ChemiDoc Imaging System.

### Immunofluorescence microscopy of BORF2-A3B bodies

HEK293T cells seeded on poly(lysine)-treated coverslips were co-transfected at 80% confluency with 1 µg of pcDNA-HA-A3B and 1 µg of either pCMV3F-BORF2 IPAM, pCMV3F-BORF2 K741R, pCDNA-BORF2 472, or pCMV3F, or 0.5 µg pCMV3F-BORF2 using linear polyethylenimine (Polysciences, Inc.) according to the manufacturer’s recommendations. In some experiments, transfections were performed with 0.5 µg pCMV3F BORF2 in the absence of pcDNA-HA-A3B. After 48 hours, cells were washed in PBS, fixed in 4% methanol-free formaldehyde (Sigma-Aldrich) in PBS for 20 minutes, rinsed twice with PBS, and permeabilized with 1% Triton X-100 in PBS for 5 minutes, then blocked overnight in 4% BSA in PBS. Cells were incubated with primary rabbit anti-HA (Cell Signaling Technology; C29F4; 1:200) and mouse anti-FLAG (Sigma-Aldrich; F1084; 1:1,000) in 4% BSA in a humid chamber for 1 hour. Cells were rinsed with PBS and incubated with secondary antibodies, goat anti-rabbit Alexa Fluor 488 (Thermo Fisher Scientific; 1:700) and goat anti-mouse Alexa Fluor 647 (Thermo Fisher Scientific; 1:700), in 4% BSA in a humid chamber for 1 hour. Cells were rinsed with PBS and mounted with ProLong Gold Antifade Mountant containing DAPI (Thermo Fisher Scientific). Images were captured using a 63× oil objective on a Leica inverted fluorescence microscope and analyzed using Leica Application Suite X software. A total of 50–100 cells were counted (as indicated in the figure legends) from approximately four microscopy fields.

For experiments in AGS cells, AGS cells on poly(lysine)-treated coverslips were transfected at 80% confluency with 2 µg of either pCMV3F-BORF2 IPAM, pCMV3F-BORF2 K741R, pCDNA-BORF2 472, or pCMV3F, or 1 µg pCMV3F-BORF2 using linear polyethylenimine according to the manufacturer’s recommendations, then fixed and blocked as described above. Cells were then incubated with primary rabbit anti-A3B ([26]; 1:100) and mouse anti-FLAG (Sigma-Aldrich; F1084; 1:1,000) in 4% BSA in a humid chamber overnight, followed by secondary antibody treatment and imaging as described above.

### Immunofluorescence microscopy of BORF2-A3B bodies in the context of EBV infection

For experiments examining SUMO staining of BORF2-A3B bodies, AGS-EBV-Z cells seeded on poly(lysine)-treated coverslips were reactivated to the lytic cycle at 80% confluency by incubation with 2 µg/mL doxycycline for 18 or 24 hours, then fixed and blocked as above. Cells were stained with primary rabbit anti-A3B ([26]; 1:100), mouse anti-BORF2 (85K BORF2 antibody [27]; 1:200), and, where indicated, sheep anti-SUMO2/3 (a gift from Ronald T. Hay [28]; 1:200) in 4% BSA in a humid chamber overnight. After rinsing in PBS, cells were incubated with secondary antibodies donkey anti-mouse Alexa Fluor 488 (Thermo Fisher Scientific; 1:700), donkey anti-rabbit Alexa Fluor 555 (Thermo Fisher Scientific; 1:700), and donkey anti-sheep Alexa Fluor 647 (Thermo Fisher Scientific; 1:700) in 4% BSA in a humid chamber for 1 hour, then imaged as described above.

To compare BORF2 mutants in the context of EBV infection, AGS-EBV-B2KO cells on poly(lysine)-treated coverslips were transfected at 80% confluency with 4 µg of pCMV3F-BORF2 IPAM or pCMV3F-BORF2 K741R, 2 µg pCDNA-BORF2 472, or pCMV3F, or 1 µg pCMV3F-BORF2 using Lipofectamine 2000 transfection reagent (Invitrogen) according to the manufacturer’s recommendations. After 8 hours, cells were reactivated to the lytic cycle by transfecting 2 µg of pBS-BZLF1 (25) using Lipofectamine 2000 transfection reagent according to the manufacturer’s recommendations. After 18 or 24 hours, cells were fixed and blocked as above, then stained in a humid chamber overnight with primary rabbit anti-FLAG (Cell Signaling Technology; D6W5B; 1:200) and mouse anti-EBV ZEBRA (BZLF1) (Santa Cruz Biotechnology, sc-53904; 1:200, 18 hours timepoint) or mouse anti-EBV EA-D (BMRF1) (Santa Cruz Biotechnology, sc-58121, 24 hours timepoint), followed by a 1 hour incubation in a humid chamber with secondary antibodies donkey anti-mouse Alexa Fluor 488 (Thermo Fisher Scientific; 1:700) and donkey anti-rabbit Alexa Fluor 647 (Thermo Fisher Scientific; 1:700). For experiments that included co-staining for A3B, cells were stained 24 hours post-reactivation with rat anti-FLAG (Thermo Fisher Scientific; MA1-142; 1:200), mouse anti-EBV EA-D (BMRF1), and rabbit anti-A3B, followed by secondary antibodies donkey anti-rabbit Alexa Fluor 488 (Thermo Fisher Scientific; 1:700), donkey anti-mouse Alexa Fluor 555 (Thermo Fisher Scientific; 1:700), and goat anti-rat Alexa Fluor 647 (Thermo Fisher Scientific; 1:700). Cells were imaged as described above.

### Immunofluorescence microscopy of ORF61-A3B and UL39-A3B bodies

HEK293T cells seeded on poly(lysine)-treated coverslips were transfected at 80% confluency with 1 µg of either pCMV3F-ORF61 or pCMV3F-UL39 with or without 1 µg of pcDNA-HA-A3B, using linear polyethylenimine (Polysciences, Inc.) according to the manufacturer’s recommendations. After 48 hours, cells were fixed and stained for FLAG and HA as described above. AGS cells were similarly transfected at 80% confluency with 2 µg of pCMV3F-ORF61 or pCMV3F-UL39, and 48 hours later were fixed and stained for FLAG.

### ProteoStat assay

HEK293T cells seeded on poly(lysine)-treated coverslips were transfected at 80% confluency with 1 µg of pcDNA-HA-A3B and 1 µg of either pCMV3F-BORF2, pCMV3F-ORF61, or pCMV3F-UL39 using linear polyethylenimine (Polysciences, Inc.) according to the manufacturer’s recommendations. Where indicated, pcDNA-HA-A3B was omitted, and cells were transfected with 2 µg of either pCMV3F-ORF61 or pCMV3F-UL39. After 48 hours, cells were fixed and blocked, then incubated with mouse anti-FLAG and rabbit anti-HA (samples transfected with pcDNA-HA-A3B only), followed by goat anti-mouse Alexa Fluor 647 and goat anti-rabbit Alexa Fluor 488 (samples transfected with pcDNA-HA-A3B only) as described above. Cells were then stained with ProteoStat aggresome detection reagent (prepared according to the manufacturer’s instructions) in a humid chamber for 30 minutes, then rinsed with PBS and mounted with ProLong Gold Antifade Mountant containing DAPI (Thermo Fisher Scientific). Images were captured using a 63× oil objective on a Leica inverted fluorescence microscope and analyzed using Leica Application Suite X software. The positive control for ProteoStat staining was conducted by treating 293T cells with 10 µM MG-132 for 18 hours, followed by fixing and staining with ProteoStat aggresome detection reagent as described above.

## SUPPLEMENTAL MATERIAL

Fig. S1 jvi.00693-25-s0001.tif Detection of lytically infected cells with BZLF1 and BMRF1 antibodies.

ASM does not own the copyrights to Supplemental Material that may be linked to, or accessed through, an article. The authors have granted ASM a non-exclusive, world-wide license to publish the Supplemental Material files. Please contact the corresponding author directly for reuse.
