# Supplementary figures and images for: Identification of Epstein-Barr virus BORF2 sequences required for APOBEC3B relocalization
Source: J Virol. 2025 Aug 21;99(9):e00693-25. doi: 10.1128/jvi.00693-25 (PMC12455968; doi:10.1128/jvi.00693-25)

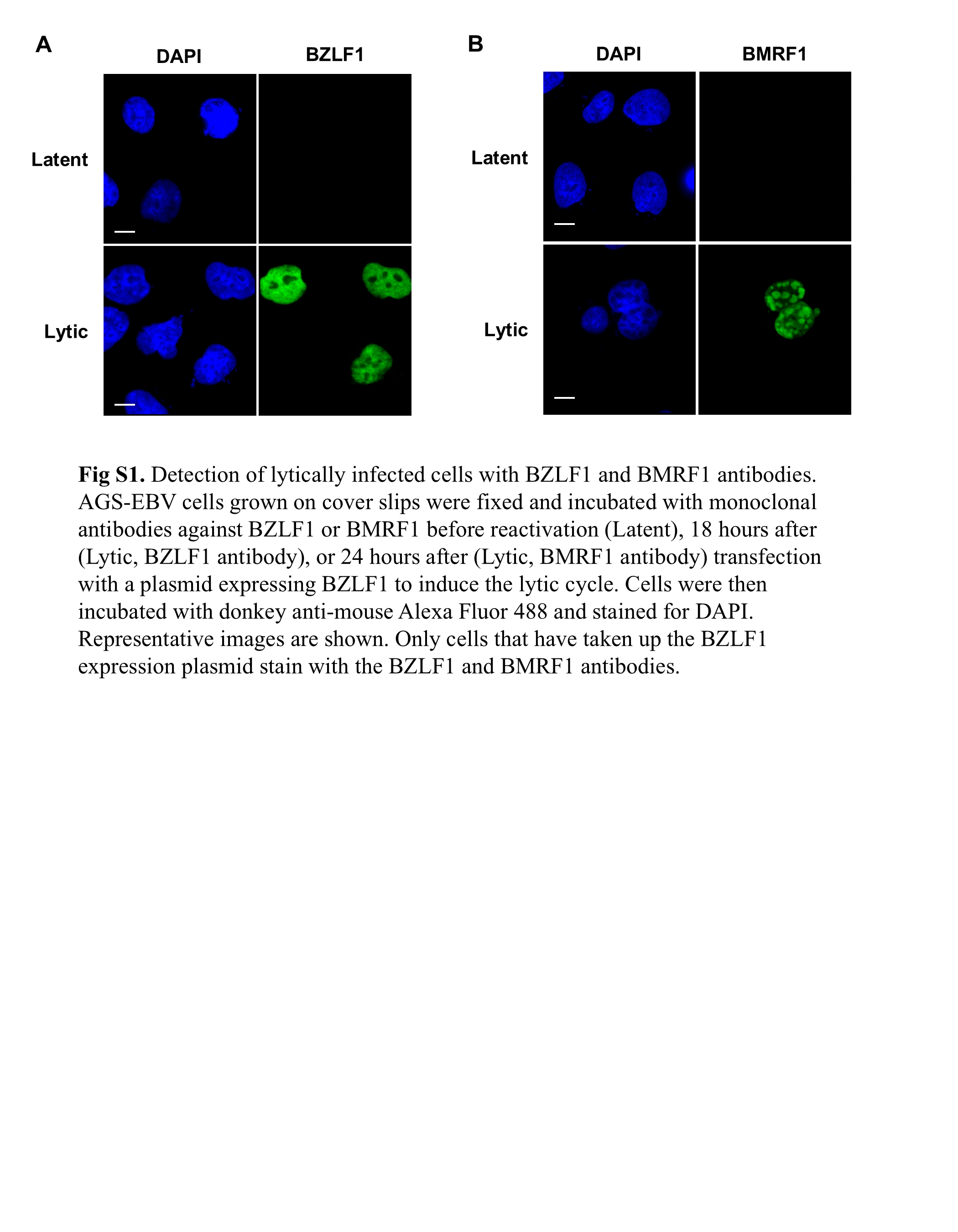

Supplement: Fig. S1 — Detection of lytically infected cells with BZLF1 and BMRF1 antibodies. [file jvi.00693-25-s0001.tif]
